# The Polio Vaccination Status of Non-polio Acute Flaccid Paralysis Cases in the Far North Region of Cameroon: A Five-Year Retrospective Study From 2015 to 2019

**DOI:** 10.7759/cureus.52740

**Published:** 2024-01-22

**Authors:** Blaise Wakam Nkontchou, Etienne Guenou, Collins Buh Nkum, Celine Mairousgou Tchida, Alphonse Marie Nono, Jerome Ateudjieu

**Affiliations:** 1 Epidemiology and Public Health, Ministry of Public Health, Yaoundé, CMR; 2 Epidemiological Surveillance Section, National Public Health Laboratory, Ministry of Public Health, Yaoundé, CMR; 3 Health Research, Meilleur Accès aux Soins de Santé (M.A. SANTE), Yaoundé, CMR; 4 Public Health, Faculty of Medicine and Biomedical Sciences, University of Yaoundé, Yaoundé, CMR; 5 Regional Centre for Epidemic Prevention and Control, Far North Regional Public Health Delegation, Ministry of Public Health, Maroua, CMR; 6 Epidemiology and Public Health, World Health Organization, Maroua, CMR; 7 Public Health, Faculty of Medicine and Pharmaceutical Sciences, University of Dschang, Dschang, CMR; 8 Division of Health Operations Research, Cameroon Ministry of Public Health, Yaoundé, CMR

**Keywords:** far north region cameroon, immunization, surveillance, acute flaccid paralysis (afp), polio

## Abstract

Background: The Expanded Program on Immunization (EPI) of Cameroon contributes to the reduction of polio, but the rate of non-polio acute flaccid paralysis (NPAFP) is still high. The aim of this study was to describe the immunization profile of NPAFP cases and the performance of polio surveillance in the Far North Region of Cameroon between 2015 and 2019.

Methods: A retrospective secondary data analysis was conducted using the national EPI and regional AFP surveillance case-based database from 2015 to 2019. Analyses were carried out using Epi-Info statistical software (version 7) (Centers for Disease Control and Prevention, Atlanta, GA).

Results: The surveillance network of the region reported 848 cases of NPAFP between 2015 and 2019. The sex distribution of the AFP cases revealed that 43.3% were females and 56.7% were males. Cases with AFP aged less than five years accounted for the largest proportion of cases (67.2%). Overall, 733/848 (86.4%) of the AFP cases received at least three doses of the oral polio vaccine (OPV). The AFP detection rate substantially increased in the region after the introduction of community-based surveillance in 2016. The mean NPAFP level during the study period was 7.3/100,000 children aged less than 15 years. The mean proportion of AFP cases with two adequate stools was 668/848 (78.7%), and the mean proportion of stools to the national reference laboratory within three days was 466/848 (54.9%).

Conclusion: Only 86.4% of AFP cases received three or more doses of OPV required for immunization. The stool specimen management indices were not good enough to confirm that no case of poliovirus was missed in the laboratory. To strengthen the country’s polio-free status, surveillance should be strengthened in least-performing health districts to improve the quality of AFP case investigations after detection.

## Introduction

In 1988, at the 41st World Health Assembly in Geneva, Switzerland, a resolution was adopted to eradicate poliomyelitis [1]. The Global Polio Eradication Initiative (GPEI) was initiated with four strategies to implement eradication activities: maintaining high-level immunity against polio in the population; detecting and interrupting the transmission of all cases of poliomyelitis through sensitive acute flaccid paralysis (AFP) surveillance; organizing Supplemental Immunization Activities (SIAs); and completing multiple campaigns [1,2]. Five of the six regions in the World Health Organization (WHO), including the African region, region of the Americas, South-East Asia Region, European Region, and the Western Pacific Region are already certified as polio-free, while the Eastern Mediterranean Region is not polio-free. The final stage of eradication seems to be more challenging because of the antivaccine campaigns and the emergence of AFP cases due to the circulation of derived poliovirus in many African countries [3,4]. Of the 200 poliovirus infections, approximately one person will suffer irreversible paralytic disease - 1 per 200 to 1 per 1000 cases - for a case fatality rate (CFR) of 5% to 10%. Poliomyelitis has no cure and can be prevented only by vaccination [1].

Several performance indicators have been developed for monitoring the performance (sensitivity and quality) of AFP surveillance programs [5,6]. However, two key indicators are most commonly used for monitoring the sensitivity and quality of AFP surveillance. The non-polio acute flaccid paralysis (NPAFP) rate should be ≥ 2/100000 children younger than 15 years, and the proportion of AFP cases with adequate stool specimens should be ≥ 90% [6]. Good stool conditions mean that the specimen reached the national laboratory within 72 hours of collection without desiccation or leakage, with evidence of reverse cold chain maintenance during transportation and appropriate documentation [7].

Cameroon belongs to the African region of the WHO and was certified "free of wild poliovirus circulation" (WPV) by the Africa Regional Certification Commission (ARCC) in June 2020 at its 25th Ordinary Meeting [8]. The ARCC is the independent body responsible for monitoring and overseeing the certification process in the African region and the only body mandated to certify the polio-free African region [9]. However, in 2016, four cases of WPV and several cases of the circulating vaccine strain poliovirus (cVDPV2) were confirmed in the State of Borno in Nigeria, which shares a long border with Cameroon [10]. Since then, several polio emergency plans have been developed to strengthen surveillance for AFP and increase the collective immunity of the population against poliomyelitis. Postresponse assessments to the 2016 outbreak revealed shortcomings in terms of the organization of AVS, routine vaccination, and epidemiological surveillance, which demonstrates the existence of a residual risk of circulation of WPV in the Far North region [10].

Because of the weakness of the EPI programme, AFP surveillance has been strengthened mainly in priority health districts through the implementation of community-based surveillance. Outbreaks of cVDPV2 remain frequent in Cameroon and in the neighbouring countries of Chad and the Central African Republic [6]. Thus, the country must establish and maintain an efficient clinical and environmental surveillance system to detect and investigate all cVDPV2 epidemics in a timely manner and to organize the response. The surveillance system operates at the central, regional and health facilities where surveillance staff are deployed [11]. AFP surveillance is the cornerstone of successful polio eradication programmes, as it helps to monitor the effectiveness of intervention strategies and can also help to identify populations in which poliovirus is transmitted [12].

Knowing the vaccination status of AFP cases and describing the country’s AFP surveillance system is highly important. Many cases of AFP have been reported in the past five years; numerous stools reach the reference laboratory after 72 hours, and some stools received in the laboratory are in poor condition. This weakness in the investigation phase can make laboratory diagnosis difficult. This is why the vaccination status of each AFP case should be known. Few studies have been conducted on NPAFP cases and their surveillance system performance in the country, but unfortunately, no studies have been carried out with the aim of determining the vaccination status of non-polio-AFP cases in Cameroon. For this reason, the present study aimed to describe the AFP surveillance system, determine the key epidemiological features of NPAFP cases and identify the vaccination profile of AFP cases according to the accessibility of their residence and the type of population.

## Materials and methods

Design

We conducted a retrospective review of routinely collected data on AFP cases in the far northern region of Cameroon between 2015 and 2019 using secondary data. These secondary data on AFP surveillance for the reporting period were found in the database of the national EPI direction and the regional EPI services. Data on sociodemographic characteristics and the doses of the polio vaccine received were extracted. These data were distributed according to the reporting health districts, according to the activities from which the cases were reported, according to the year they were reported, and according to the type of surveillance actors that reported the AFP cases. All the data for each AFP case were checked for errors and poor or missing information.

Study setting

The study was conducted in the Far North Region of Cameroon. It shares international borders with the Republic of Chad in the east, the Federal Republic of Nigeria in the west and a national border with the North region. The Far North is a region at high risk for poliovirus transmission and was the first region in the country to face a cVDPV2 outbreak in May 2019. The region has 30 health districts. The total estimated population of the region for 2019, based on a growth rate of 2.5%, was 4,728,412, with children younger than 15 years representing approximately 50.65% of the population. The database of the immunization services of the regional delegation for the health of the Far North Region of Cameroon and that of the national EPI central group were the main places for data collection. Routinely collected data from the AFP investigation forms at the regional delegation for public health will be reviewed.

Study population

The AFP data were collected from children younger than 15 years of age living in or visiting the Far North Region. The use of AFP for all confirmed or compatible polio cases was excluded from the study. The study population included all reported NPAFP cases from 2015 to 2019.

Sampling

The target population of the study included all NPAFP cases reported and investigated from 2015 to 2019. All cases with NPAFP levels that were reported in the Far North Region between 2015 and 2019 were included in the sample.

Data collection

The traditional AFP questionnaire was developed by integrating the sociodemographic characteristics of the participants through several variables, such as age, sex, place of residence, type of population and vaccination history. The data were collected from an electronic database where all variables on the investigation forms of the reporting period were stored. This database was secured and available at the national EPI permanent secretariat at Yaoundé. The distribution of AFP cases according to sex, age, year of reporting, reporting health district, number of oral polio vaccines (OPVs) received, reporting type of surveillance, quality of persons who detected the AFP cases, and activities during which AFP cases were discovered.

Data analysis

The data collected from the database were analysed using descriptive and inferential statistics. The goal of obtaining descriptive statistics was to accurately describe all the responses to a variable. Descriptive statistical analysis was used to describe and summarize the variables using tables and graphs. As the results of the study are intended to be used by EPI officials, the data analysis used the simplest possible statistics to present the results of the study and to make them clear and accessible. The main outcomes measured in our study were the proportion of AFP cases who received at least three doses of the OPV, the annual NPAFP rate for under 15 children, the proportion of stool specimens reaching the laboratory within 72 hours, the number of adequate samples and the impact of community-based surveillance on AFP surveillance activities. The independent variables for this study were age, sex, year, health district and region in the Far North. We could not obtain data on certain outcomes, such as type of population and residence, because variables were not available in the database.

Ethical considerations

Ethical approval for the study was obtained from the Cameroon National Ethics Committee for Research on Human Health. Permissions were also given by the National EPI permanent secretary and the regional delegate for public health of the Far North Region to use the EPI data available in their database [13]. Ethical approval was also obtained from the University of Roehampton. Verbal permission was obtained from the data managers at the national and regional levels. We protected the confidentiality of the AFP cases. The names and identifiers of the participants were not extracted or stored in this study, and there was no risk to privacy or confidentiality [14].

## Results

Sociodemographic characteristics

During the study period from 1st January 2015 to 31st December 2019, a total of 848 AFP cases were reported through the surveillance system network. A total of 78% of the AFP cases were verified by a member of the health district board before the investigation was performed, after which the stool specimens were sent to the national reference laboratory. Four age categories were considered in the study, and we evaluated children younger than one year, children aged between one and five years, children aged between six and 15 years and children aged older than 15 years. Cases with AFP aged less than five years accounted for the largest proportion of cases (67.21%), followed by those aged 6-15 years (25,58%). In 37 (4.36%) of the AFP cases, age was unknown or was missing on the investigation forms. The sex distribution of the AFP cases revealed that 43.27% were females and 56.72% were males (Table 1).

**Table 1:** Trends and key epidemiological features of AFP cases in the Far North Region of Cameroon, 2015-2019 AFP: acute flaccid paralysis

Year	Annual Number of AFP Cases	Sex		Age (Years)				
		Male n(%)	Female n(%)	< 1 n(%)	1-5 n(%)	6-15 n(%)	˃15 n(%)	Unknown/missing
2015	89	50 (56.18%)	39 (43.82%)	0 (0%)	59 (66.26%)	22 (24.72%)	8 (8.99%)	0 (0%)
2016	141	88 (62/41%)	53 (37.59%)	3 (2.13%)	78 (55.32%)	48 (34.04%)	12 (8.51%)	0 (0%)
2017	195	112 (57.44%)	83 (42.56%)	11 (6.64%)	134 (68.72%)	46 (23.59%)	4 (2.05%)	0 (0%)
2018	247	132 (53.44%)	115 (46.56%)	9 (3.67%)	172 (70.20%)	64 (26.12%)	0 (0%)	2 (0.80%)
2019	176	99 (56.25%)	77 (43.75%)	3 (2.13%)	101 (71.63%)	37 (26.24%)	0 (0%)	35 (19.88)
Overall	848	481 (56.72%)	367 (43.27%)	26 (3.06%)	544 (64.15%)	217 (25.58)	24 (2.83%)	37 (4.36%)

All 30 health districts in the Far North Region reported AFP cases within the five years of study, with the majority from health districts implementing community-based surveillance. In health districts where community-based surveillance activities are implemented, 529/848 (62.38%) AFP cases were reported, with only 44.25 children younger than 15 years. For example, in health districts with no AFP community base surveillance, only 329/848 (37.61%) AFP cases were diagnosed, although a high proportion of those under 15 years of age were diagnosed (55.75%). A total of 96.66% of the health districts in the region achieved the target NPAFP rate during the study period. Less sensitive AFP surveillance in the Far North Region was performed in the following health districts: Maga, Moulvoudaye and Kar-Hay. Maga is the only health district with an overall annual mean NPAFP concentration less than the recommended 3/100,000 children younger than five years (Table 2). At least one case of AFP was reported in all the health districts during the study period of five years. Roua, Goulfey, Bourrha and Mora are health districts where AFP surveillance is very sensitive, with 12.68%, 12.68%, 11.73% and 10.85%, respectively, of the annual non-polio-AFP detection rate. These rates were far above the minimum recommended by the WHO.

**Table 2:** Distribution of AFP in health districts in the Far North Region AFP: acute flaccid paralysis, NPAFP: non-polio acute flaccid paralysis

	Health Districts of the Far North Region	Population ≤ 15 years 2019	Annual Number of AFP Cases Reported					Total AFP Cases	Overall NPAFP Rate
			2015	2016	2017	2018	2019		
Health districts with AFP community-based surveillance activities									
1	HINA	66629	2	5	7	13	9	36	10.81
2	MOGODE	62722	3	12	10	5	3	33	10.52
3	MOKOLO	146315	17	5	12	8	14	56	7.65
4	MORA	149280	2	7	28	35	9	81	10.85
5	MAROUA 2	100700	6	8	12	18	8	52	10.33
6	GOULFEY	48915	0	5	4	14	8	31	12.68
7	KOLOFATA	59623	1	0	4	15	3	23	7.72
8	KOUSSERI	219441	7	16	16	27	16	82	7.47
9	KOZA	89848	2	3	2	11	8	26	5.79
10	MADA	81144	1	3	2	10	11	27	6.65
11	MAKARY	99486	0	3	9	7	9	28	5.63
12	ROUA	48908	0	5	7	7	12	31	12.68
13	BOURHA	39223	1	7	4	4	7	23	11.73
	Total 1	1212234	42	79	117	174	117	529 (62.38%)	8.73
Health districts without AFP community-based surveillance activities									
14	GUERE	68246	3	3	3	6	2	17	4.98
15	BOGO	59472	2	4	9	6	5	26	8.74
16	GAZAWA	37616	2	4	7	1	3	17	9.04
17	GUIDIGUIS	89425	4	4	8	7	3	26	5.81
18	KAELE	58197	2	3	3	4	3	15	5.15
19	KAR HAY	67279	4	1	2	1	5	13	3.86
20	MAGA	97654	2	2	2	3	2	11	2.25
21	MAROUA 1	97446	3	4	2	12	12	33	6.77
22	MAROUA 3	88226	4	6	7	6	1	24	5.44
23	MERI	84308	0	1	4	4	9	18	4.27
24	MINDIF	29009	2	1	4	3	3	13	8.96
25	MOULVOUDAYE	74776	0	6	3	3	1	13	3.48
26	MOUTOURWA	27529	1	4	4	1	1	11	7.99
27	PETTE	30975	0	1	4	2	2	9	5.81
28	TOKOMBERE	73818	4	3	4	4	2	17	4.61
29	VELE	76109	4	5	5	4	4	22	5.78
30	YAGOUA	123751	5	10	7	6	1	29	4.69
	MISSING							5	
	Total 2	1183836	42	62	78	73	59	319 (37.61%)	5.39
	TOTAL 1+2	2396070	89	141	195	247	176	848	7.37

Vaccination status of cases with AFP

Overall, 733/848 (86.42%) of the AFP cases received at least three doses of OPV and were supposed to be immunized against polio because they had a good immune system at the time the OPV was given. A total of 40/848 (4.72%) received 1-2 doses, while 29/848 (3.42%) never received OPV. The polio immunization status of 4.83% of the AFP cases was unknown because respondents were not able to remember the number of doses children had received. In 2015, only 64.04% of the AFP cases received at least three doses of the polio vaccine (Table 3). In 2018, 94.74% of AFP cases were vaccinated with at least three doses of the OPV. The reasons for this increase in OPV coverage are probably the launch of the country’s response to an outbreak in the Far North Region due to the cases of four wild polioviruses and several cases of cVDPV2 reported in 2016 in Borno State, Nigeria, which shares a long border with Cameroon.

**Table 3:** Trends and key epidemiological features of AFP cases in the Far North Region of Cameroon, 2015-2019 AFP: acute flaccid paralysis

Year	Annual AFP Cases	Total Oral Polio Vaccine Doses				
		0 n(%)	1-2 n(%)	3+ n(%)	Unknown n(%)	Missing n(%)
2015	89	9 (10.11%)	10 (11.24%)	57 (64.04%)	9 (10.11%)	4 (4.50%)
2016	141	4 (2.84%)	6 (4.26%)	112 (79.43%)	18 (12.7%)	1 (0.71%)
2017	195	9 (4.62%)	9 (4.62%)	166 (85.13%)	11 (5.64%)	0 (0%)
2018	247	4 (1.62%)	6 (2.42%)	234 (94.74%)	3 (1.21%)	0 (0%)
2019	176	3 (1.70%)	9 (5.11%)	164 (93.18%)	00 (0%)	0 (0%)
Overall	848	29 (3.42%)	40 (4.72%)	733 (86.42%)	41 (4.83%)	5(0.59%)

Activities during which AFP cases were reported

As shown in Table 4, 824/848 (97.17%) AFP cases were detected by routine surveillance, while 24/848 (2.83%) were detected during mass campaign immunizations. A total of 211/848 (24.88%) AFP cases were diagnosed by surveillance community agents, 606/848 (71.46%) by health workers, and 13/848 (1.53%) by other sources. The reporting sources of all AFP cases were known. SIAs offer opportunities for the active identification of AFP cases and are usually used for case detection. Community-based surveillance of AFP boosted surveillance both in the community and in health facilities. From 84 cases of AFP in 2016, the total number of AFP cases reported in the Far North Region every year reached 244 in 2018.

**Table 4:** Repartition of AFP cases according to activity and case status AFP: acute flaccid paralysis, SIA: Supplemental Immunization Activities

Year	Annual Number of AFP Cases	Activities During Which AFP Cases Were Discovered		Quality of Persons Who Detected the AFP Cases		
		Routine surveillance n(%)	SIA n(%)	Community Health agents n(%)	Health workers n(%)	Others n(%)
2015	89	89(100%)	0(0%)	0(%)	84(100%)	0(0%)
2016	141	141(100%)	0(0%)	23(16.31%)	111(78.72%)	7(4.96%)
2017	195	177(90.77%)	18(9.23%)	32(16.41%)	161(82.56%)	2(1.03%)
2018	247	246(99.60%)	1(0.40%)	99(40.08%)	145(58.70%)	2(0.81%)
2019	176	171(97.16%)	5(2.84%)	57(32.39%)	105(59.66%)	2(1.14%)
Overall	848	824(97.17%)	24(2.83%)	211(24.88%)	606(71.46%)	13(1.53%)

AFP surveillance indicators

A review of the surveillance indicators for polio eradication in the region from 2015 to 2019 showed that no cases of WPV were reported in the region within that period. Table 5 shows the evolution of the AFP performance indicators in the Far North Region during the period of study. The most important AFP indicator, the NPAFP rate, which measures the sensitivity of the system, reached the target value in 2015, 2016, 2017, 2018 and 2019. The annual NP-AFP concentration during the study period was 2.87 per 100,000 children under 15 years of age, 4.1/100,000 in 2015, 6.3/100,000 in 2016, 8.5/100,000 in 2017, 10.5/100,000 in 2018 and 7.3/100000 in 2019 (Table 5). Since 2016, there has been a gradual increase in the total number of AFP cases reported every year as well as the annual NPAFP rate because of the beginning of the AFP community-based surveillance implemented in some health districts in the region, as shown in Table 2. AFP-related cases were reported in all 30 health districts of the Far North Region within the five-year period of the study. Overall, 668/848 (78.77%) were reported within ≤14 days of paralysis onset, while 180/848 (21.22%) were reported after ≥14 days of paralysis onset. The transportation of AFP-treated stool specimens revealed that 466/848 (54.95%) of the stool specimens arrived at the national reference laboratory within 72 hours of collection. A total of 382/848 (55.05%) arrived at the laboratory after 72 hours. Between 2015 and 2019, 790/848 (93.16%) of the stool specimens were judged to be in good condition at arrival at the national reference laboratory. The proportion of AFP cases whose stool specimens were judged to be in good condition at the reference laboratory was consistently above the minimum target of 80% in five consecutive years. In 2016, all major surveillance indices were achieved, with an annual NPAFP rate of 6.33/100,000 children under 15 years of age, 80.14% of AFP cases with two stools collected within 14 days after the onset of paralysis and 93.61% of stool to the laboratory within three days.

**Table 5:** AFP surveillance indicators 2015-2019 AFP: acute flaccid paralysis, NPAFP: non-polio acute flaccid paralysis

Year	Pop 2019 ≤15 Years	Annual Number of AFP Cases Reported	Annual NPAFP Rate	AFP Cases With Two Stools Collected Within 14 Days After Paralysis n(%)	Stool to Lab Within Three Days n(%)	Stool Reaching Lab in Good Conditions n(%)
Target			≥3	80%	90%	80%
2015	2,170,684	89	4.10	67 (75.28%)	84 (94.38%)	84 (94.38%)
2016	2,224,951	141	6.33	113 (80.14%)	132 (93.61%)	134 (95.03%)
2017	2,280,575	195	8.55	153 (78.46%)	77 (39.48%)	177 (90.76%)
2018	2,337,589	247	10.56	197 (79.75%)	67 (27.12%)	223 (90.28%)
2019	2,396,070	176	7.34	138 (78.40%)	106 (66.22%)	172 (97.72%)
Overall		848	7.37	668 (78.77%)	466 (54.95%)	790 (93.16%)

Clinical history of AFP cases

Fever at the onset of paralysis was observed in 747/848 (88.08%), sudden onset of paralysis was reported in 578/848 (68.16%) and 281/848 (33.13%) had asymmetric paralysis (Table 6).

**Table 6:** Clinical characteristics of the AFP cases AFP: acute flaccid paralysis

Year	Number of AFP Cases	Signs and Symptoms		
		Fever	Sudden onset of paralysis	Asymmetrical paralysis
2015	89	84 (94.38%)	17(19.10%)	70 (78.65%)
2016	141	138 (97.87%)	42(29.78%)	121(85.81%)
2017	195	173 (88.71%)	156(80%)	15(07.69%)
2018	247	236 (95.54%)	218(88.25%)	38(15.38%)
2019	176	116 (65.90%)	145(82.38%)	37(21.02%)
Overall	848	747 (88.08%)	578(68.16%)	281(33.13%)

## Discussion

The present study aimed to highlight the vaccination status of cases with NP AFP and to describe the performance of AFP surveillance in the Far North Region of Cameroon between 2015 and 2019. The results of the study indicated that the surveillance of AFP was very sensitive during the study period, so the GPEI/WHO minimum annual NP AFP rate was always exceeded. The results of this study revealed that a substantial number of AFP cases were not immunized against poliomyelitis disease and that there was a sensitive polio surveillance system evidenced by high AFP detection and NP AFP detection rates during the study period. To maintain the country’s polio-free polio circulation, a highly sensitive surveillance system is needed, and no case of AFP that could have been caused by WPV or cVDPV should be missed.

Sociodemographic characteristics

The main reason for evaluating the age distribution was that age was a risk factor for poliomyelitis disease in children younger than five years. Four age categories were included in the study: younger than one year, one to five years, six to fifteen years and older than fifteen years. Cases with AFP aged less than five years accounted for the largest proportion of cases (67.21%). The sex distribution of AFP cases revealed that 56.72% were males. A similar proportion of males was observed in a study conducted in the DRC, in which male children aged less than 5 years represented approximately 55% of the AFP Cases [15].

This proportion of children under five years of age was lower than that reported in similar studies in Akwa Ibom State-Nigeria [16], India, Ghana [5] and Kenya [1], with 94.4%, 90%, 76.3% and 63.6%, respectively, and a proportion of children under five years of age reported. In the Democratic Republic of the Congo (DRC), among the 13,749 AFP cases analysed, the largest proportion (85.2%) were children under five years of age [15]. A lower proportion (58.7% and 37.0%) of children under five years of age were also reported in Amritsar, India [17], and the Marches region, Italy [18], in similar studies. Bassey et al. suggested that the reasons for these variations may be attributed to surveillance sensitivity and the active or passive type of surveillance practised by health facilities.

Most of the AFP cases included in the study were male (56.72%), unlike in the studies conducted in Akwa Ibom State-Nigeria [16] and in Ido Ekiti-Nigeria [19], in which most AFP cases were female. Other surveys conducted by D’Errico et al. [18] in Italy reported a greater incidence of AFP among boys than among girls. In another study in Nigeria, 56% of AFP cases were males [5].

This study revealed the effectiveness of community-based surveillance for detecting and reporting additional cases of AFP. Community-based surveillance was implemented in more than 30 of the 12 health districts where more AFP cases were detected (529/848; 62.38%) than in health districts with no AFP community-based surveillance, where only 329/848 (37.61%) AFP cases were reported. Community-based AFP surveillance was called for to rescue weak health systems that were not able to produce the annual minimum number of AFP cases demanded by GPEI/WHO directives. Previously, there was no involvement of the community in the surveillance network, and all AFP cases were reported by health facilities. With community-based surveillance, community health agents, traditional healers, traditional rulers, matrons and even medicine vendors were sensitized, trained and integrated into the surveillance system. There are currently more reported AFP cases than reported by health facilities.

The mean annual NP AFP concentration in health districts that perform community-based surveillance was 8.7 for 100000 children aged less than 15 years. In health districts without AFP community-based activities, the prevalence was 5.3%. This is an indication of the strong involvement of the community in the surveillance network. The community surveillance network is composed of health community agents and community informants. The health community workers are recruited, trained, supervised and motivated with the help of NGOs that finance AFP surveillance activities. Community health agents also perform household visits and conduct campaigns to increase awareness of poliomyelitis. They motivate parents and community informants (community leaders) to report early suspected cases of AFP in their households or their neighbourhood [20].

Vaccination status of AFP cases

A total of 86.42% of the AFP cases in the study received at least three doses of OPV and were considered immunized. The remaining 13.38% were considered not immunized. The proportion of AFP cases who received at least three doses of OPV was similar to the 82.5% of AFP cases who received at least three doses of OPV in a similar study conducted in Akwa Ibom State, Nigeria [16]. Almost the same proportion (88.1%) of AFP cases received three or more doses of OPV in a study conducted in Kenya [1]. In Akwa Ibom State, Nigeria, a lower proportion of AFP cases (60.3%) 534/885 received more than >3 doses of OPV, while 196/885 (22.2%) received three doses of OPV, and 128/885 (14.5%) received between one and two doses of OPV [16]. A study of DRC titres showed that only 58.9% of AFP cases had received at least three doses of OPV.

Low immunization coverage of OPV by the population may result in serious consequences in countries that use OPV. Among these consequences are outbreaks of poliomyelitis due to cVDPVs (CDC, 2010). The number of OPVs received by AFP cases might have been underestimated or overestimated by recall bias. Sometimes parents or the caregivers who responded to the investigators’ questions were not present or informed each time OPV doses were given to the children. Sometimes, during the investigation of AFP cases, the immunization status of most children cannot be traced because of the absence of immunization cards [5,21]. The high polio immunization coverage observed is attributable to many rounds of mass vaccination campaigns against poliomyelitis. These supplementary immunization activities against polio were in the form of follow-up or catch-up campaigns or in response to the poliomyelitis outbreak. Twenty-five rounds of mass immunization campaigns against polio were organized in the Far North Region between 2016 and 2019.

Activities during which AFP cases were reported

In our study, we were interested in the reporting sources of AFP cases and the quality or identity of the persons who detected and reported cases. This approach may help in evaluating the importance of these biomarkers in the AFP surveillance system. A total of 211/848 (24.88%) AFP cases were diagnosed by surveillance community agents, 606/848 (71.46%) by professional health workers, and 13/848 (1.53%) by other sources. Most of the AFP cases (506/848; 59.67%) were reported in the 12 health districts where community-based surveillance was implemented. The remaining 342 (40.33%) AFP cases were from the remaining 18 health districts where there is no AFP community-based surveillance. Unfortunately, similar studies in which we reviewed publications did not show any interest in the origin of AFP cases or the quality of the reported persons.

AFP surveillance indicators

The surveillance indicators that we were able to evaluate were the annual NP AFP concentration, two stools collected within 14 days after paralysis, one stool sample reaching the national reference laboratory within three days and one stool sample reaching the national laboratory in good condition. During the study period, Cameroon was considered a polio outbreak country, and the incidence of AFP was expected to be at least ≥ 3 per 100,000 children aged <15 years. The results of the study during the period revealed that AFP surveillance was efficient according to the NP AFP test results, which are far above the three standards for 100,000 children aged <15 years old recommended by the WHO. Additionally, approximately 80% of the AFP cases had two stools collected within 14 days after paralysis, and the proportion of stool specimens reaching the national laboratory in good condition was always above 90%. The mean annual NP AFP concentration in the region during the study period was 7.37/100,000 children younger than 15 years of age. However, the distribution of the sensitivity indicators for the mean annual NP AFP concentration varied with Maga not meeting the target throughout the five-year period. The mean NP AFP level was above 2.87/100,000 in Kenya in another study [1].

The proportion of stool samples that reached the national reference laboratory within three days was 94.3% in 2015. This proportion started decreasing shortly after AFP community-based surveillance was introduced in some health districts in 2016. This introduction was followed by massive reports of AFP cases that could not be properly investigated by few available investigators, adding to the difficulties of sending the numerous resulting stool samples to the national laboratory within three days. As the number of AFP cases increased due to community-based surveillance, the proportion of cases whose stool reached the national laboratory within three days decreased over the years. The overall proportion of stool specimens reaching the national laboratory within three days of the study was 54.95%. Overall, 668/848 (78.77%) AFP cases were diagnosed within ≤14 days of paralysis onset. This proportion fell below the target of 80%. Bassey et al. [4], in a similar study in Nigeria, reported 84% of the overall 743/885 AFP cases within ≤14 days of paralysis onset, while Brook et al., in a similar study in Kenya, found that only two adequate specimens were collected for 1512 (88.6%) AFP cases within 14 days [1].

Clinical history of AFP cases

Our study revealed that a greater proportion of the children with fever at the onset of paralysis (747/848 [88.08%]; 578/848 [68.1%]; and 281 [33.1%]) had sudden onset of paralysis. Similar findings were obtained in other surveys. In a study conducted by Bassey et al. [16], 797/885 (90.1%) cases were found to have a fever at the onset of paralysis, and paralysis was found to be asymmetric in 805/885 (91%). In the DRC, 82.7% of cases had fever at the onset of paralysis, 94.7% of the AFP cases had sudden onset of paralysis, and 42.1% had asymmetric paralysis [15]. Our results are also similar to those found in 2018 in Kenya by Brook et al. [1], who reported that 1099 (64.5%) AFP cases had fever at the onset of paralysis, and sudden onset of paralysis was reported in 904 (53.0%) of the AFP cases.

Other authors had different results. Sevencan et al. [22] reported that 15% of AFP cases had fever at disease onset in a similar study in Turkey. Saeed and al. [23] reported that sudden onset of paralysis occurred in 28% of cases and that asymmetrical paralysis occurred in 67% of cases. A study performed in western India after eight years of AFP surveillance showed that 21.6% of AFP cases had fever at disease onset and 22 (24.7%) had asymmetric paralysis [24].

Limitations

We registered limitations mainly at the level of the database where data were missing. Some AFP case investigation forms were not completely filled. The recall bias had a negative effect on the number of doses each AFP case received.

## Conclusions

Our study revealed that polio surveillance has positive points but also reveals some existing gaps that need to be identified and bridged to improve polio surveillance indicators. AFP surveillance was sensitive during the five years covered by the study, and the target of three or more AFP cases for a population of 100,000 children under five years of age was always reached. The sensitivity and quality of AFP surveillance decreased with time, particularly after the country was declared polio-free, reflecting the loss of awareness about poliomyelitis. Cases of AFP are rare, and health community workers are less proactive because of the nonpayment of their parents. The performance of the annual NP AFP rate was always greater than three for 100,000 children younger than 15 years. The introduction of AFP community base surveillance in 2026 was followed by an exponential increase in the number of cases reported mostly in the health districts where it was implemented.

The proportion of AFP cases who received the required three doses of OPV to be immunized against polio was 86.42%. The remaining 13.58% of AFP cases were considered not immunized and could be a source of virus transmission. More AFP cases were reported in health districts where community-based surveillance activities were implemented than in those without community-based surveillance. The indicators used to measure stool management performance were not good, although 93.16% of the stool samples that reached the reference laboratory were in good condition. Many community health workers were hired for AFP community-based surveillance in many health facility catchment areas. In each health facility catchment area, only one AFP, an already busy health centre officer, was investigated. The ratio of investigative officers to community health workers is approximately 1/10, which could also explain why the AFP levels were not sufficient. Therefore, strengthening the investigation component of the AFP surveillance system, particularly in terms of stool specimen handling, is necessary to ensure that the system is capable of demonstrating the absence of transmission of poliovirus in the region. Adequate and continuous training of the community health workers, community informants and officers in charge of AFP investigation and sensitization of clinicians will improve the quality of AFP surveillance in the Far North Region.
